# Chemical and Structural Stability of Lithium-Ion Battery Electrode Materials under Electron Beam

**DOI:** 10.1038/srep05694

**Published:** 2014-07-16

**Authors:** Feng Lin, Isaac M. Markus, Marca M. Doeff, Huolin L. Xin

**Affiliations:** 1 Environmental Energy Technologies Division, Lawrence Berkeley National Laboratory, Berkeley, CA 94720, USA; 2 Department of Materials Science and Engineering, University of California, Berkeley, CA 94720, USA; 3 Center for Functional Nanomaterials, Brookhaven National Laboratory, Upton, NY 11973, USA

## Abstract

The investigation of chemical and structural dynamics in battery materials is essential to elucidation of structure-property relationships for rational design of advanced battery materials. Spatially resolved techniques, such as scanning/transmission electron microscopy (S/TEM), are widely applied to address this challenge. However, battery materials are susceptible to electron beam damage, complicating the data interpretation. In this study, we demonstrate that, under electron beam irradiation, the surface and bulk of battery materials undergo chemical and structural evolution equivalent to that observed during charge-discharge cycling. In a lithiated NiO nanosheet, a Li_2_CO_3_-containing surface reaction layer (SRL) was gradually decomposed during electron energy loss spectroscopy (EELS) acquisition. For cycled LiNi_0.4_Mn_0.4_Co_0.18_Ti_0.02_O_2_ particles, repeated electron beam irradiation induced a phase transition from an 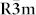 layered structure to an 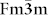 rock-salt structure, which is attributed to the stoichiometric lithium and oxygen removal from 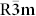 *3a* and *6c* sites, respectively. Nevertheless, it is still feasible to preserve pristine chemical environments by minimizing electron beam damage, for example, using fast electron imaging and spectroscopy. Finally, the present study provides examples of electron beam damage on lithium-ion battery materials and suggests that special attention is necessary to prevent misinterpretation of experimental results.

Lithium-ion batteries are used in a variety of consumer electronics, such as mobile phones, tablets and laptops, and are now being commercialized for use in electric vehicles (EVs) and plug-in hybrid electric vehicles (PHEVs)^1^,^2^,^3^,^4^. In order to achieve reliable battery packs for these applications, various thrusts of research and development must be conducted simultaneously, including but not limited to the design of advanced battery materials, cell configuration, circuit optimization, and diagnostics. With respect to the chemistries of electrode materials, various analytical diagnostic tools have been performed to probe chemical and structural environments from the bulk to the surface^5^,^6^,^7^,^8^,^9^. In-depth diagnostic analysis can provide insights into structure-property relationships^10^,^11^ as well as into failure mechanisms^12^,^13^. In combination with many ensemble-averaged measurements, fast electron microscopy and spectroscopy are poised to reveal chemical and structural information with extremely high spatial resolution and potentially improved temporal resolution through *in situ* transmission electron microscopy (TEM) visualization^14^,^15^. To date, a vast number of studies have been successfully implemented by virtue of the state-of-the-art imaging and spectroscopy capabilities of fast electron microscopy.

The chemical environment at the particle surface determines the way an active electrode interacts with its surrounding electrolyte and impacts the resulting cycling behaviors. Surface modifications, such as artificial solid-electrode interphases, were reported to improve cycling performance^16^,^17^. On the other hand, electrochemistry-induced surface reaction layers (SRLs), such as solid-electrolyte interphases at anode material particle surfaces, also play critical roles in determining the way electrode materials interact with electrolytic solutions and how lithium ions intercalate^18^. SRLs are generally composed of oligomers and inorganic compounds that are formed from the electrochemical reduction of solvent and electrolytic salt^19^. Similar to the behavior of many organic compounds under electron beams, SRLs are prone to beam damage. Structural changes may also occur as a result of charge-discharge cycling, and have been proven to impact battery performance. For example, in LiNi_x_Mn_x_Co_1−2x_O_2_ (NMC), lithium-rich Li(Li_y_Ni_x−y_Mn_x_Co_1−2x_)O_2_, lithium-rich/manganese-rich xLi_2_MnO_3_**·**(1 − x)LiMO_2_ (M = Mn, Ni, Co, etc.) materials, the transition from layered structures to spinel and/or rock-salt structures leads to impedance buildup, voltage decay, and capacity fading^5^,^12^,^20^,^21^. The referenced studies were performed following *ex-situ* TEM procedures, and the electrochemical performance was successfully correlated to the phenomena observed using TEM. Lithium-containing compounds (e.g., electrode materials, SRLs) are easily degraded under electron beams due to the knock-on effect (i.e., atomic displacements by electron-nuclear collisions)^22^ and thermal effects. It is therefore, critical to distinguish these processes from those arising from the electrochemistry to ensure the correct interpretation of the experimental results.

The present work was designed to understand the effect of electron beam damage on surface reaction layers and NMC materials during data acquisition using TEM. It is shown that the chemistry and structure can be severely altered under certain circumstances, such as low scan rates. The degradation processes resemble the lattice reconstruction and chemical evolution that are caused by charge-discharge cycling^5^. It is suggested that reducing the accelerating voltage and increasing acquisition speed (e.g., performing fast EELS) are necessary to avoid the beam damage.

## Results

**Figure 1:**
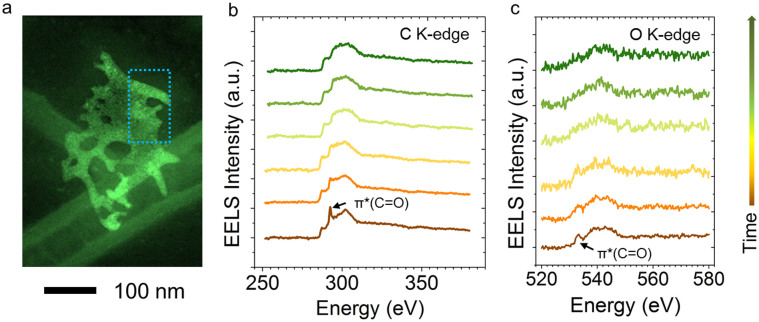
Stability study of Li_2_CO_3_ phase on a lithiated NiO nanosheet. (a) Annular dark-field scanning transmission electron microscopy (ADF-STEM) image with an area identified for EELS acquisition. (A 200 keV electron beam (~12 pA) rapidly scanned the area repeatedly to spread the dose during EELS acquisition.) (b) C K-edge EELS spectra. (c) O K-edge EELS spectra. Each spectrum was integrated for 5 sec with a dose rate of 100 electrons/Å^2^/sec and an accumulated dose of 500 electrons/Å^2^. The critical dose limit for lithium carbonate is estimated to be approximately 750 electrons/Å^2^ placing it in sensitivity next to biomolecules (<200 electrons/Å^2^ at 77 K)^32^. The arrow to the right of (c) indicates the increased acquisition time from the bottom to the top in (b) and (c), with an interval of 5 sec.

The identification of the chemical environment of SRLs (e.g., solid-electrolyte interphases) is essential to understanding their functionalities in the battery operation^19^. Owing to its high spatial resolution, STEM-EELS is believed to be one of the most advantageous techniques for this purpose. Carbonate groups have been shown to dominate at the surfaces of many cathode and anode materials. Figure 1 shows EELS measurements on a lithiated NiO nanosheet. In a previous study, we showed that, in NiO nanosheets, the SRLs are primarily composed of Li_2_CO_3_ embedded in a complex organic matrix^6^. Indeed, the fingerprints for the 1s to π* transition of the CO_3_^2−^ group are simultaneously observed in the C K-edge and O K-edge spectra, as shown in Figure 1b and Figure 1c, respectively. The well-defined characteristic peaks were gradually degraded after repeated EELS acquisition over the area indicated by the dashed boxed in Figure 1a, and the fine structures of the CO_3_^2−^ group disappeared after four acquisitions. The compounds in the SRLs are usually prone to thermal decomposition^23^. Here we showed that the combined knock-on, ionization, and thermal effects of the electron beam readily broke down the surface structure leading to the degradation of the SRL. The damage can be mitigated by acquiring EELS data from a larger area while keeping the dose below the critical limit. In addition, the critical dose limit (measured in electrons/Å^2^) is generally dependent on the dose rate (measured in electrons/Å^2^/sec). By decreasing the dose rate (i.e. reducing the incident beam current while keeping the scanning area constant), the damage rate can potentially be reduced^24^.

**Figure 2:**
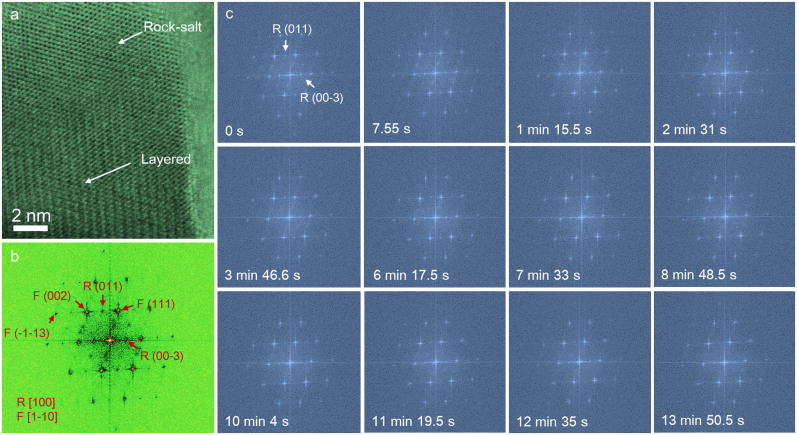
Bright-field STEM (BF-STEM) observation of an NMC-based composite particle under electron beam irradiation (300 keV, ~36 pA, 1024 × 1024 pixels, pixel size 0.153 angstrom, pixel dwell time 6 us, frame time 7.55 sec/frame). (a) BF-STEM image of an NMC particle coated with rock-salt (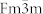) structure at the surface. (b) The corresponding fast Fourier transform (FFT) of the BF-STEM image in (a), where the zone axes for the 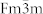 and 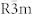 phases are [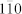] and [100], respectively. (c) FFT results for a series of BF-STEM images under electron beam irradiation, where the FFT results correspond to pristine (0 s), 1^st^ frame (7.55 s), 10^th^ frame (1 min 15.5 s), 20^th^ frame (2 min 31 s), 30^th^ frame (3 min 46.6 s), 50^th^ frame (6 min 17.5 s), 60^th^ frame (7 min 33 s), 70^th^ frame (8 min 48.5 s), 80^th^ frame (10 min 4 s), 90^th^ frame (11 min 19.5 s), 100^th^ frame (12 min 35 s) and 110^th^ frame (13 min 50.5 s) in the series. The intensity of the diffraction spots for the 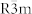 phase decreased during exposure to the electron beam. F and R represent the 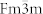 and 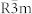 phases in the FFT indices, respectively. The corresponding BF-STEM and FFT movies are provided in Movie S1 and Movie S2 in the supplemental information, respectively.

A focused electron beam was scanned over a LiNi_0.4_Mn_0.4_Co_0.18_Ti_0.02_O_2_ (abbreviated as NMC hereafter) particle that had undergone 20 electrochemical cycles between 2.0–4.7 V *vs.* Li^+^/Li, ending in the discharged state. There are two dominant crystal structures found in the particle; 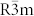 and 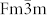 (Figure 2a). The 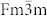 rock-salt structure was generated by lattice reconstruction of 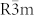 (filling of 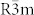 *3a* sites by transition metals) as a result of oxygen loss and concomitant lithium ion removal during cycling^5^,^21^,^25^. In Figure 2b, Fast Fourier transform (FFT) of the high-resolution bright-field STEM (BF-STEM) image (Figure 2a) show distinct diffraction spots for the 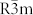 and 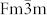 crystal structures along the zone axes of 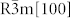 and 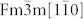, respectively. During electron beam irradiation, the 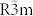 structure gradually converted to the 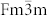 structure, as recorded in a BF-STEM mode movie (Movie S1). Selected FFT patterns shown in Figure 2c give evidence of the conversion of the 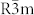 structure. Two characteristic diffraction spots, i.e., R(011) and R(00-3), are indicated in the patterns and their intensities were monitored during the irradiation process. Ultimately, after an extended irradiation period (13 min 50.5 sec, 110^th^ frame), these two diffraction spots lost their intensities completely. The superimposed FFT patterns are presented in Movie S2.

**Figure 3:**
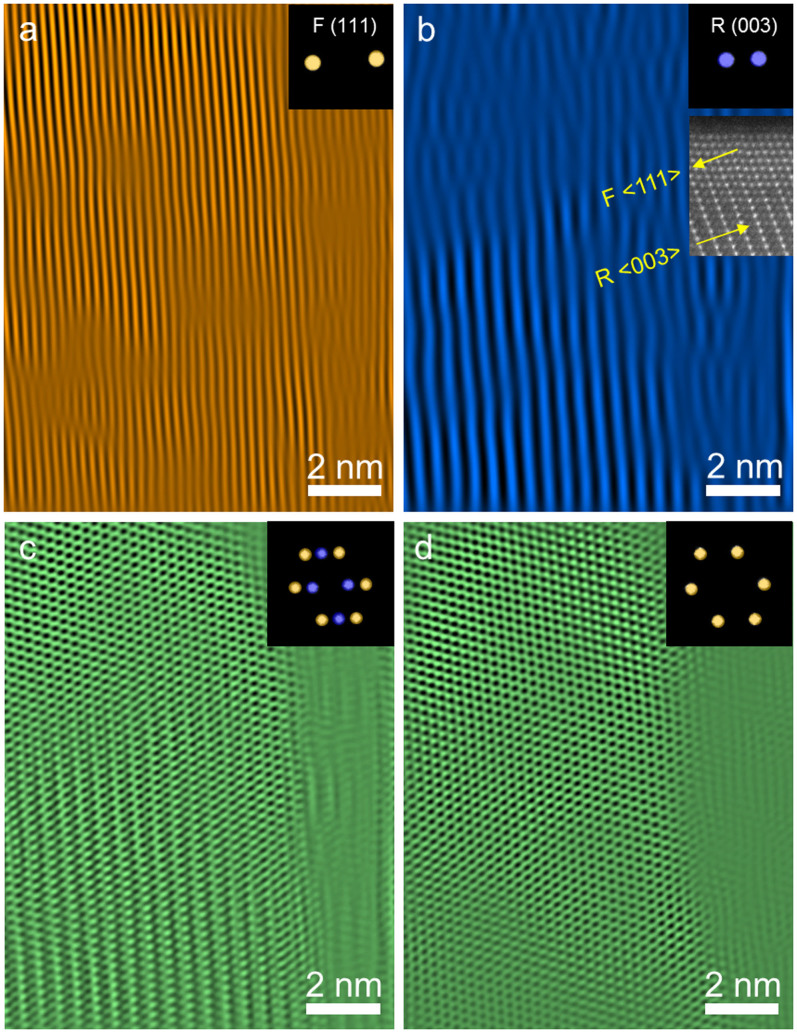
Inverse FFT images of an 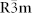/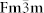 composite particle obtained by placing masks on the FFT patterns. (a) Inverse FFT image of 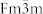 (111) planes before electron beam irradiation. (b) Inverse FFT image of 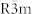 (003) planes before electron beam damage, and the ADF-STEM image in the inset shows an example for the orientation relationship between the 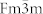 and 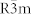 structures. (c) Inverse FFT image of the 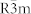/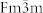 composite particle before electron beam damage. (d) Inverse FFT image of the 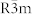/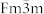 composite particle after electron beam irradiation. The diffraction spots masked for inverse FFT imaging are shown in the insets, where the purple and yellow spots correspond to 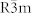 and 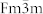 phases, respectively.

The crystal orientation relationship between the 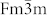 and 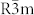 structures is visualized by inverse FFT imaging, as shown in Figure 3. The 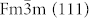 and 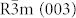 facets are selectively visualized by masking the corresponding diffraction spots in the FFT pattern (Figures 3a and 3b, and the insets therein). The lattice spacing of the 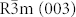 of the NMC phase is approximately twice as large as that of the 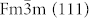 spacing of the rock-salt phase (MO, M = Ni, Mn, Co); i.e., there is ~5% of mismatch, so that the rock-salt phase grew epitaxially on the layered phase. The inset of Figure 3b exemplifies the epitaxial growth of 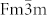 on 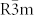 in real space. The inverse FFT images were generated for the 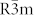/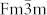 composite particle before (Figure 3c) and after (Figure 3d) electron beam damage. The initial 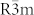/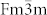 composite particle (Figures 3c) was completely converted to a pure 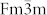 phase after electron beam damage (Figure 3d). The phase transition process likely proceeded *via* transition metals moving from *3b* sites to *3a* sites, resulting in the collapse of the layered structure and the formation of a pure rock-salt structure.

**Figure 4:**
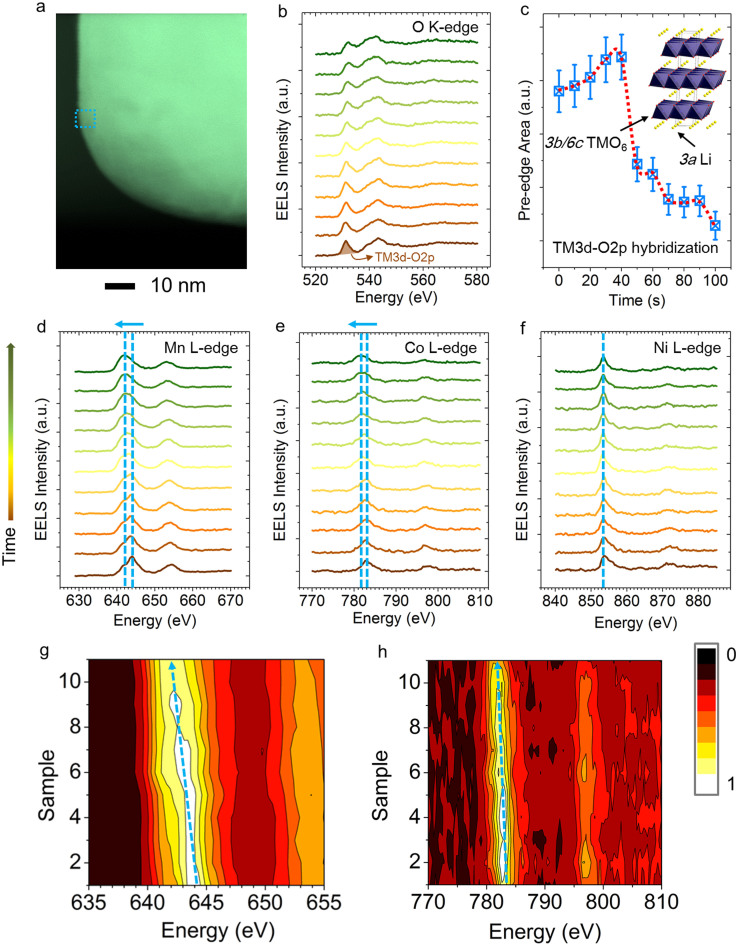
EELS study of an NMC particle using a continuous acquisition mode. (a) STEM image of the particle with the selected region for EELS data collection. (b) O K-edge EELS spectra. (c) O K-edge pre-edge integrated intensity as a function of electron beam irradiation time. The pre-edge intensity corresponds to TM*3d*-O*2p* hybridization states in the *3b* sites, as shown in the inset. The integrated pre-edge intensity is in a positive relationship with the oxidation states of TMs in the TMO_6_ octahedral units. (d–f) TM L-edge spectra corresponding to the O K-edge spectra in (b), where the shifts of L_3_ peaks are indicated by the dashed lines and arrows. The arrow to the left of (d) indicates the increased exposure time from the bottom to the top in (d), (e) and (f), with an interval of 10 sec. (g, h) 2D EELS maps enhanced for visualization of the peak shifts for Mn (g) and Co (h). Each spectrum was integrated for 10 sec with a dose rate of 113,000 electrons/Å^2^/sec and an accumulated dose of 1,130,000 electrons/Å^2^. It shows that the critical dose limit for NMC material is approximately 6,000,000 electrons/Å^2^, which is four orders of magnitude higher than that of lithium carbonate.

The requirement for simultaneous lithium and oxygen removal during the 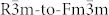 transition implies that the transition metals are reduced during the process. The changes in the oxidation state of transition metals (TMs) were monitored by EELS and are shown in Figure 4. The transition metal L-edge EELS measures the dipole allowed transitions from metal 2*p* orbitals to unoccupied metal 3*d* orbitals, including both the 2*p_3/2_* (L_3_) and 2*p_1/2_* (L_2_) spin-orbit final states, and indirectly probes the local hybridization states for metal-oxygen octahedral units (i.e., TMO_6_) in NMC materials^26^,^27^,^28^. The O K-edge EELS corresponds to the transition from O*1s* states to unoccupied O2*p* states in the conduction band. Due to the TM3*d*-O2*p* hybridization, O K-edge spectra also reflect the unoccupied TM3*d* states^29^. Although the exact nature of the hole states (e.g., location) depend on the degree of covalency in the TMO_6_ octahedral unit, it is generally agreed that, in the (Ni, Mn, Co)-O_6_ octahedral unit, the intensity of the pre-edge of the O K-edge is in a positive relationship with the formal oxidation states of (Ni, Mn, Co) due to the sharing of hole states in the TMO_6_ unit^30^. A continuous data acquisition mode was used to collect EELS spectra on a selected region shown in Figure 4a. As the acquisition proceeded, the electron beam gradually damaged the NMC particle. There was an incubation period from 0–40 s in the integrated pre-edge intensity before it dropped dramatically at 50 s, indicating that an accumulation of lattice interruptions by electron beam was necessary to initiate the removal of lithium and oxygen. TM L-edge EELS investigation provided more direct evidence for the changes in the oxidation states of transition metals, as shown in Figures 4d–h. The formal oxidation states of Ni, Mn and Co in the pristine NMC structure are +2, +4 and +3, respectively^5^. The most salient features of the TM L-edge spectra can be captured by the peak positions of TM-L_3_ edges. As the acquisition proceeds (from the bottom to the top in Figures 4d, 4e, 4g and 4h), there are noticeable peak shifts towards lower energy for Mn-L_3_ and Co-L_3_ edges, indicating that Mn and Co were both gradually reduced. As expected, Ni remained in the +2 oxidation state in the 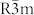 and 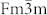 structures. The synergistic changes in the oxidation states of oxygen and transition metals suggest that the hole states are shared by the transition metals and oxygen in the TMO_6_ unit.

**Figure 5:**
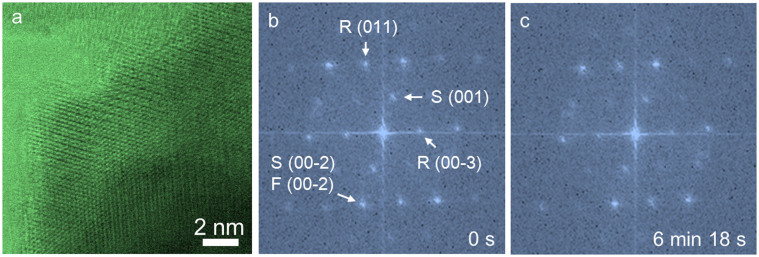
BF-STEM observation of an 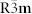/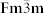/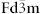 composite particle under electron beam with a 4× faster frame rate (300 keV, ~36 pA, 512 × 512 pixels, pixel size 0.306 angstrom, pixel dwell time 6 us, frame rate 1.89 sec/frame). (a) BF-STEM image of a particle edge region with similar thickness and crystal orientation relative to that of Figure 2a. (b) FFT pattern of the composite particle before electron beam damage, where the rock-salt, spinel and layered structures are observed in the pattern. Note that the 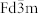 phase is an intermediate during the 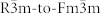 transition in electrochemical cycles^5^,^21^. (c) FFT pattern of the composite particle after electron beam damage, which corresponds to the 200^th^ frame (6 min 18 s) in the series. The corresponding BF-STEM movie is provided in the supplemental information (Movie S3). F, R and S represent the 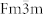, 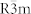 and 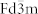 phases in the FFT indices, respectively.

Decreasing the dose rate is critical in reducing radiation damage because isolated bond breaking is healable in conductive solid samples^24^,^31^. Rastering the beam over an area is an efficient way to reduce the dose rate. If the beam is idle, the dose rate is the beam current normalized by the beam area. If the beam is scanned sufficiently fast, the dose is spread out over the scanning area; in this case, the dose rate is the beam current normalized by the entire scanning area. To test how scan rate affect the dose rate, we performed a parallel experiment to compare with the condition used in Figure 2. As shown in Figure 5, we preserved the beam current, the field of view, and the dwell time but reduced the pixel number by a factor of four (i.e. scanning was four times faster). When the beam damage was compared under these two conditions after similar number of frames (i.e., similar repeat of electron beam scanning), it shows that the structural damage was significant less than that in Figure 2. In the 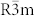/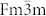/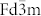 composite particle (Figure 5a), the 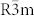 phase was preserved even after extended number of frames (compare Figures 5b and 5c). This suggests that ultrafast scanning in conjunction with drift correction is desirable for STEM imaging of radiation sensitive battery materials.

## Discussion

We have shown that electron beam damage is a common phenomenon affecting battery materials studied by TEM. For example, the Li_2_CO_3_ component in the SRL of a lithiated NiO nanosheet rapidly decomposed during EELS data acquisition. NMC cathode materials also degraded at low scan rates or with intensive doses. Li K-edge EELS energy onset is buried between the M-edges of transition metals in NMC cathode materials, while transition metal oxidation states are also prone to change. These intrinsic characteristics make the direct evidence of lithium removal unlikely using Li K-edge EELS during irradiation. Nevertheless, during electron beam irradiation, the formal oxidation states of Mn and Co were reduced from +4 to +2 and from +3 to +2, respectively. Therefore, correlating the present *in situ* BF-STEM and EELS results with previous studies^5^,^21^, one can determine that the degradation process involved simultaneous lithium and oxygen removal from the *3a* and *6c* sites of the 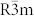 structure, and transition metals moving from the *3b* sites to *3a* sites, resulting in the collapse of the layered structure and ultimate conversion to a 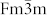 structure. The degradation process is similar to the lattice reconstruction observed in several classes of layered cathode materials, including NMC, lithium-rich Li(Li_y_Ni_x−y_Mn_x_Co_1−2x_)O_2_, and lithium-rich/manganese-rich xLi_2_MnO_3_**·**(1 − x)LiMO_2_ (M = Mn, Ni, Co, etc.) materials. It is also likely that the electron beam reduces transition metals first due to its strong reducing characteristics and then knocks out and/or evaporates lithium and oxygen from the NMC material, eventually leading to transition to an 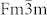 structure. Furthermore, due to the thermodynamic instability of charged NMC materials^5^, similar degradation is supposed to occur in the charged NMC particles. The present study suggests that, under some circumstances, the lattice reconstruction induced by electron beams can be used as a model system to study structural dynamics in electrochemical processes. Finally, special caution is recommended in studying battery materials to avoid indiscriminate sample degradation. This can be achieved by increasing the irradiation area and the scan rate.

## Methods

### Materials synthesis and battery cycling

The syntheses of NiO and LiNi_0.4_Mn_0.4_Co_0.18_Ti_0.02_O_2_ (NMC) were performed according to previously developed protocols^5^,^6^. In short, NiO was synthesized using a solvothermal method aided with an alcohol pseudo-supercritical drying technique. NMC was synthesized using a co-precipitation method followed by high-temperature annealing with LiOH. 2032 Coin cells were fabricated using composites of NiO or NMC as working electrodes and lithium metal foils as counter electrodes. The NiO working electrodes were composed of 80 wt.% active material, 10 wt.% polyvinylidene fluoride (Kureha Chemical Ind. Co. Ltd) and 10 wt.% acetylene carbon black (Denka, 50% compressed) and loadings were typically 1–2 mg/cm^2^ of active material. To make the electrodes, these solids were mixed into N-methyl-2-pyrrolidinone and the resulting slurry cast onto copper current collectors and dried. NMC working electrodes were prepared similarly and contained 84 wt.% active material, 8 wt.% polyvinylidene fluoride, 4 wt.% acetylene carbon black and 4 wt.% SFG-6 synthetic graphite on carbon-coated aluminum current collectors, with typical active material loadings of 6–7 mg/cm^2^. The coin cells were assembled in a helium-filled glove box using Celgard 2400 separators and 1 M LiPF_6_ electrolyte in 1:2 w/w ethylene carbonate/dimethyl carbonate (Ferro Corporation). Battery testing was performed on a computer controlled VMP3 potentiostat/galvanostat (BioLogic). NiO and NMC electrodes were cycled at C/2 and C/20 rates, respectively. 1C was defined as fully discharging or charging an electrode in 1 h, corresponding to specific current densities of 718 mA/g and 280 mA/g for NiO and NMC materials, respectively.

### Electron microscopy and spectroscopy

For electron microscopy and spectroscopy measurements, the electrode particles were scratched off and deposited onto TEM grids after the desired number of electrochemical cycles. A 200 keV and a 300 keV field-emission (scanning) transmission electron microscope (S/TEM) were used for *in situ* imaging and spectroscopic studies. The 200 keV microscope was operated with an imaging condition of 11 mrad, 12 pA. The 300 keV instrument was operated with 17 mrad, 36 pA. Electron energy loss spectroscopy data sets were acquired using Gatan Tridiem spectrometers.

## Author Contributions

All authors participated in conceiving the work. F.L. designed, prepared the materials and performed electrochemistry with the assistance from I.M.M. H.L.X. performed electron microscopy and spectroscopy. F.L. and H.L.X. designed and analyzed the electron microscopy and spectroscopy experiments. F.L. prepared the figures and wrote the manuscript with the assistance from all authors. M.M.D. and H.L.X. participated in supervising the work. All authors reviewed the manuscript.

## Supplementary Material

Supplementary Information Movie S1

Supplementary Information Movie S2

Supplementary Information Movie S3
